# Supplementary material for: Hepatitis C virus seroprevalence, testing, and treatment capacity in public health facilities in Ghana, 2016–2021; A multi-centre cross-sectional study
Source: PLoS One. 2023 Jun 23;18(6):e0287580. doi: 10.1371/journal.pone.0287580 (PMC10289322; doi:10.1371/journal.pone.0287580)
Supplement: Attachment — Submitted filename: Response to reviewers PLOS HCV 230523.docx [file pone.0287580.s002.docx]

Department of Internal Medicine and Therapeutics

Cape Coast Teaching Hospital

P.O.BOX CT1363

Cape Coast

Ghana

22^nd^ May 2023

The Academic Editor,

PlosOne

Dear Academic Editor,

Response to Reviewers’ Comments

Thank you for the opportunity to submit the original research article entitled “Hepatitis C virus seroprevalence, testing, and treatment capacity in public health facilities in Ghana, 2016 – 2021; A multi-centre cross-sectional study.” by Nartey et. al. with manuscript number PONE-D-23-08352 for consideration by your journal. We thank the reviewers for their review of the manuscript and insightful comments. Please find our point-by-point responses to comments raised below.

Reviewer #1:

1. The rapid test kits used in this sero-prevalence study of hepatitis C need to have an evidence of post market verification using a serological test gold standard such enzyme immuno assay or multiplex beads assay as a gold standard.
   a. We agree with the reviewer. There was variability in test kits used by study sites over the testing period studied, therefore we could not obtain information on test kit performance/verification. The pitfalls of failure to use WHO prequalified kits is discussed on page 17 line 358-360 and in the limitations on page 19 line 410-412
2. Are remnants of the donors samples archived in these blood banks in order to provide answers to some of these questions? If yes, is recommended that you compare the serologically positive samples with the nucleic acid testing or at least EIA results. The sensitivity, specificity, positive predictive vale and the negative predictive value from the evaluation/ post market verification of these rapid test kits are quite essential to the reliability of this sero-prevalence data.
   a. Thank you for your suggestion. This will be a good next step for this study; therefore, a new proposal will be written for ethical approval to undertake such a study. For the current paper, we are unfortunately unable to perform this analysis as we do not have permission to obtain any archived samples from 2016-2021.
3. Does the record in the blood banks indicate the route of transmission of hepatitis C among the positive donors? This will help in prevention of Hepatitis C infection in Ghana.
   a. The records in the blood-bank made available to us did not contain routes of transmission among positive donors. We are currently conducting a study to investigate the risk factors associated with HCV seropositivity in Ghana.

Reviewer #2:

1. As a Medical Laboratory Scientist , I enjoyed every bit of the article. The research methodology and data analysis was brilliant. But I beg different will that 17% data available would not be able to give a good representation of the prevalence of HCV infection in Northern Ghana. It is a brilliant idea, I hope this findings from this research is submitted to relevant stakeholders to help formulate policies that build a good disease surveillance for JCV infection in Ghana
   a. Thank you for your review and contribution.

Reviewer #3:

1. It will be of great benefit to the manuscript/audience if the authors could provide information on any HCV-related intervention or policies the Ghana health services have introduced during the study period. With an elimination target in view, I believe every country would have initiated some action/intervention. The data presented suggest an increase in HCV infections – this could result from no intervention being conducted or interventions not doing what they are intended to. We can only arrive at such a conclusion if we have an idea of what interventions have been put in place.
   a. Thank you for your suggestion. The authors are not aware of a specific HCV intervention undertaken during the study period. Information regarding a 2023 HCV-specific intervention by the Ghana Health Service has been included on page 18 lines 389-394.
2. To estimate prevalence, the authors rely on serological data. However, the authors also mentioned access to molecular data although on a small scale at 1-2 health facilities. Was this molecular data included in estimating the seroprevalence or most of these individuals with molecular test results were initially tested with a serological test? The authors should provide information on how the molecular test results were processed.
   a. We stated that “PCR testing for HCV RNA was only available in two centres visited, with one of these outsourcing to a private laboratory.” In both centers, this test had only become available a few months before the data collection period and even then, availability was dependent on whether there were cartridges to run the sample. There were only a handful of results available, which were too few to use for data analysis (fewer than 10 at the time of data collection). We therefore opted to use only seroprevalence data for this study. For individuals with molecular tests, it could not be ascertained whether the serological test was conducted within the same facility or upon request from a referral facility.
3. The study authors need to revise and standardize some minor aspects of the manuscript.
   For example, Materials and Method section (abstract) On page 5 line 108 the authors mentioned that the study was conducted in public health institutions (that could be interpreted as government-run facilities) but on page 6 lines 113 – 118, the private and faith-based health facilities were included in the review.
   a. The following institutions were stated: “Ghana Health Service hospitals, teaching hospitals, public health reference labs and faith-based institutions”. These facilities visited all fell under the public health space and not the private sector. No private institution was visited for data collection.

Furthermore, were the 16 admin regions grouped into 3 zones (northern, middle, and southern) from which the health facilities were selected. If yes, could the authors rephrase this section to better highlight what was done? Could the authors also provide information on the final number of facilities reviewed per zone?

a. We have rephrased the section as suggested (page 6 lines 115-120). The information on final number per zone has been updated in the results section on page 8 lines 164-165

In the Method section (~ 6 per zone = 18 but in the results section, the authors mentioned 23 health facilities. What of the blood banks)

a. A purposive sampling approach was used to select at least 2 two regional hospitals, 2 district hospitals, 1 faith-based hospital and 1 public health reference laboratory, - this makes up 6 per zone totaling 18 sites. Additionally, we gathered data from, “...all teaching hospitals located within each zone”. There are 5 teaching hospitals in the country, making up 23 health facilities as stated in the results. Out of the 23 sites approached, laboratory data was available for 17 institutions and 13 blood banks. The section has been updated for clarity (page 6 lines 115-120)

Interestingly, in the result sections the zones are not mentioned. So why did the authors adopt a zoning approach that was eventually not used? I will suggest the zoning aspect be deleted. It is of no significance.

a. Zoning was purposively done to ensure that a range of public health facilities in each part of the country were included in data collection in order to have a dataset that spanned all areas of Ghana.
b. Additionally, zoning helped to illustrate that the administrative regions in northern Ghana appeared to have a higher seroprevalence compared to the other two zones. Wording has been updated to reflect this on page 9, lines 192, 196, 287, 289. The discussion has previously discussed potential reasons for the difference in seroprevalence in Northern Ghana on page 14 lines 290-300.
4. As a study limitation, does the use of seroprevalence data overestimate the prevalence of HCV? Lines 73-78 as indicate a lower disease burden when viraemic data is considered. Seroprevalence data could reflect three possible scenarios 1) active infection 2) a resolved past infection and 3) a false positive. I will suggest the authors highlight this shortcoming in their analysis.
   a. This is well noted. The following has been added to the study limitations on page 19 lines 408-410 “Additionally, the use of seroprevalence data may overestimate the HCV prevalence, since anti-HCV positivity comprises those with active infection, resolved infection or a false positive test result.”
5. What is the added value of figure 3 that highlights cases < 18 years of age? The data presented by the authors suggest a higher burden as age increases. If a more granular analysis was required, the focus should have been on the elderly group. What do the authors wish to highlight in the <18 group that is more important for this group?
   a. We wish to highlight the HCV prevalence in the under 18 age-group since the WHO treatment guidelines were recently updated to include guidance on treating children with HCV infection.
6. Minor language corrections
   Introduction Line 78 page 4: modify “high burden of viremia.” to “high burden of viraemic persons” ;

Introduction Line 91 page 5: modify “The challenges to HCV response” to “The challenges of achieving the HCV elimination targets”

Methods section: Line 117 page 13 correct “refence” to “reference” laboratory
Include a reference for the Agyeman and colleagues study mentioned in line 255.

All the suggested modifications and corrections have been effected, thank you.

7. Discussion section: The statement of HCV knowledge among healthcare workers is at best speculative. If not supported by a reference. The statement should either be referenced or deleted if no reference is available. Lines 296-298
   a. The following sentence has been deleted:

“If the same is true, and HCV knowledge among healthcare workers in Ghana is insufficient, this may mean that healthcare workers may be less likely to screen, treat, or link HCV patients to appropriate care.”

Additional requirements from editor addressed:

1. Please ensure that your manuscript meets PLOS ONE's style requirements, including those for file naming.
   a. This has been done
2. In your Data Availability statement, you have not specified where the minimal data set underlying the results described in your manuscript can be found. PLOS defines a study's minimal data set as the underlying data used to reach the conclusions drawn in the manuscript and any additional data required to replicate the reported study findings in their entirety. All PLOS journals require that the minimal data set be made fully available. For more information about our data policy, please see <http://journals.plos.org/plosone/s/data-availability>.

"Upon re-submitting your revised manuscript, please upload your study’s minimal underlying data set as either Supporting Information files or to a stable, public repository and include the relevant URLs, DOIs, or accession numbers within your revised cover letter. For a list of acceptable repositories, please see http://journals.plos.org/plosone/s/data-availability#loc-recommended-repositories. Any potentially identifying patient information must be fully anonymized. Important: If there are ethical or legal restrictions to sharing your data publicly, please explain these restrictions in detail. Please see our guidelines for more information on what we consider unacceptable restrictions to publicly sharing data: http://journals.plos.org/plosone/s/data-availability#loc-unacceptable-data-access-restrictions. Note that it is not acceptable for the authors to be the sole named individuals responsible for ensuring data access. We will update your Data Availability statement to reflect the information you provide in your cover letter.

Data cannot be shared publicly due to confidentiality and local restrictions which prevent sharing abstracted laboratory and blood bank data. The relevant data are contained within the manuscript. Any other data are available from the Ghana Health Service and Teaching Hospitals included in this study for researchers who meet the criteria for access to confidential data.

3. Please include your full ethics statement in the ‘Methods’ section of your manuscript file. In your statement, please include the full name of the IRB or ethics committee who approved or waived your study, as well as whether or not you obtained informed written or verbal consent. If consent was waived for your study, please include this information in your statement as well.
   a. The methods section has been updated to include an ethics statement on page 7 line 151-161.
4. We note that Figures 2,3 and 4 in your submission contain [map/satellite] images which may be copyrighted…We require you to either (1) present written permission from the copyright holder to publish these figures specifically under the CC BY 4.0 license, or (2) remove the figures from your submission….Please upload the completed Content Permission Form or other proof of granted permissions as an "Other" file with your submission. In the figure caption of the copyrighted figure, please include the following text: “Reprinted from [ref] under a CC BY license, with permission from [name of publisher], original copyright [original copyright year].”
   a. The maps were created using MapChart. The terms of use have been uploaded and appropriate permission statement included in the figure captions.
5. Please review your reference list to ensure that it is complete and correct. If you have cited papers that have been retracted, please include the rationale for doing so in the manuscript text, or remove these references and replace them with relevant current references. Any changes to the reference list should be mentioned in the rebuttal letter that accompanies your revised manuscript. If you need to cite a retracted article, indicate the article’s retracted status in the References list and also include a citation and full reference for the retraction notice.
   a. Reviewed.

Yours sincerely,

Yvonne Ayerki Nartey MBChB PhD

Cape Coast Teaching Hospital/University of Cape Coast,

Cape Coast, Ghana

Email: [ynartey@uccsms.edu.gh](mailto:ynartey@uccsms.edu.gh)

Tel: +233 24 679 4065
